# A Case of Transmission of Bedaquiline- and Linezolid-Resistant Tuberculosis

**DOI:** 10.3390/ijms27114912

**Published:** 2026-05-29

**Authors:** Anastasia Ushtanit, Irina Peretokina, Ludmila Krylova, Svetlana Safonova, Alexey Filippov, Danila Zimenkov

**Affiliations:** 1 Center for High-Tech Bioeconomy, Engelhardt Institute of Molecular Biology, Russian Academy of Sciences, 119991 Moscow, Russia; 2 The Moscow Research and Clinical Center for Tuberculosis Control of the Moscow Government Department of Health, 107014 Moscow, Russia

**Keywords:** tuberculosis, transmission, bedaquiline, linezolid, drug resistance

## Abstract

Tuberculosis is one of the hardest-to-treat bacterial diseases with a high capacity to develop antibiotic resistance. The treatment scheme based on bedaquiline and linezolid was introduced in Russia in 2014 and, since 2018, has been widely used for the treatment of resistant tuberculosis. In our study of clinical *M. tuberculosis* isolates, we identified a case of a recent transmission of a strain with mutations in the genes *rv0678* and *rplC*, associated with resistance to bedaquiline and linezolid. We analyzed five isolates obtained from patient A between 2015 and 2019 after unsuccessful treatment and three isolates from patient B obtained between diagnosis in 2019 and death in mid-2020 via whole-genome sequencing and 24-loci MIRU-VNTR genotyping. During the treatment of patient A, a large spectrum of different mutations in *rv0678* developed, accompanied by an increase in bedaquiline MIC from 0.06 to 0.5 mg/L. Simultaneously, *rplC* C154R substitution emerged, leading to linezolid resistance. The isolates from patient B contained nearly the same mutation spectra as the isolates from patient A, differing in only four variants that emerged during transmission. The possible transmission event must have occurred in a public place in Moscow, since there was no evidence of direct contact between the patients. This finding confirms the worrying trend of untreatable *M. tuberculosis* strains circulating in the general population.

## 1. Introduction

The global epidemiological situation regarding tuberculosis has worsened recently with the emergence of multidrug- (MDR-TB) and extensively drug-resistant strains (XDR-TB), which are associated with significantly lower treatment success rates. The spread of resistant strains threatens to reverse the progress made in TB control, as such cases demand rapid diagnosis and personalized treatment; otherwise, these infections lead to poor outcomes and further disease transmission.

Since the (in general, successful) introduction of bedaquiline- and linezolid-containing regimens for the treatment of resistant tuberculosis in Russia [1], we have noticed a small number of cases without the expected culture conversion after three to five months [2], accompanied with increased MICs of bedaquiline and linezolid and the emergence of mutations in specific regions of the genome [3]. There is still an ongoing discussion as to whether mutations in the *rv0678* and *atpE* genes, which play a role in establishing bedaquiline resistance, as has been clearly demonstrated [4,5], are clinically significant and associated with a poor treatment outcome [6]. However, after several years of surveillance of such cases, the acquisition of true resistance to bedaquiline can be assumed [7,8,9], and the reported poor outcomes may be due to resistance of the pathogen to other drugs, particularly fluoroquinolones.

Bedaquiline resistance in clinics is relatively frequent compared to linezolid, which could be partially explained by the existence of intermediate resistance mechanisms driven by loss-of-function mutations in the *rv0678* (*mmpR5*) gene coding the efflux operon repressor [10]. Various *rv0678* mutations distributed along the whole open-reading frame emerge first, preceding the *atpE* gene coding for the bedaquiline target; these mutations have a limited spectrum [9].

Initially, drug resistance was assumed to necessarily be associated with fitness cost, decreasing the probability of spreading resistant strains [11]. However, the selection of variants with minimal cost and the subsequent acquisition of compensatory mechanisms forced the global spread of a drug-resistant pathogen [12]. To date, many studies have reported the transmission and spread of resistant tuberculosis, including resistance to the recently developed drugs bedaquiline, pretomanid, and delamanid [13,14]. Herein, we report a case of likely public-place transmission of a strain resistant to both bedaquiline and linezolid, identified via whole-genome sequencing.

## 2. Results

### 2.1. Transmission Event Revealed by Genomic Sequencing and Genotyping

When performing a routine study on the microevolution of *M. tuberculosis* during treatment via whole-genome sequencing, we identified the possible case of transmission of a strain with mutations in the *rv0678* and *rplC* genes, associated with resistance to bedaquiline and linezolid, respectively. First, a very similar pattern of mutations was identified via visual inspection of two variants files obtained from different patients, and it was further confirmed by the sequencing of several independent isolates from both cases and bioinformatic analysis. The isolates from the two patients comprised the separate cluster within the set of isolates sequenced in our Institute, and they had 1295 common variants, compared to the reference *M. tuberculosis* H37Rv genome.

**Figure 1:**
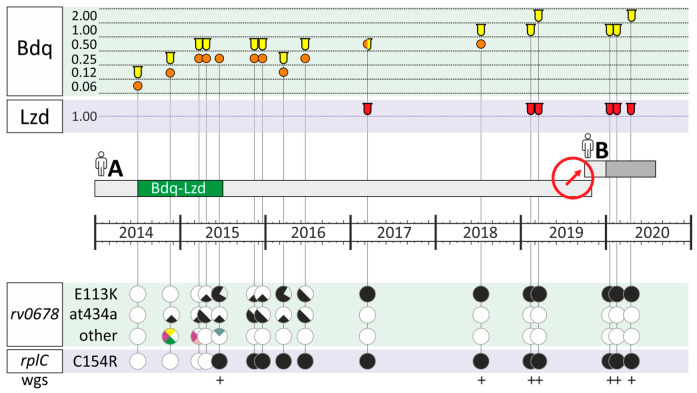
Bedaquiline and linezolid phenotype and resistance mutations in clinical isolates recovered during treatment of patients. Bedaquiline MICs were obtained via two methods using Bactec MGIT960 (BD, Franklin Lakes, NJ, USA) (yellow tubes) and agar plates (orange circles). Allelic frequencies of mutations in the *rv0678* and *rplC* genes were estimated from Sanger and whole-genome sequencing and are shown with pie charts: from 0% (white circles) to 100% (black circles). The red circle with an arrow depicts the date range of possible infection of Patient B.

Thus, we analyzed five isolates obtained from Patient A from 2015, 2018 and 2019 during relapse after unsuccessful treatment with the bedaquiline and linezolid scheme. In addition, earlier and intermediate isolates were analyzed via Sanger sequencing of *rv0678*, *atpE*, and *rplC* genes (Figure 1). Three isolates from Patient B were obtained in 2019 and the first four months of 2020. Only four positions in the genome had been changed when comparing the latest isolate from patient A and the earliest from patient B.

The isolates belong to the Central Asian/Russian sublineage of the Beijing lineage, and 24-loci MIRU-VNTR analysis confirmed the identity of isolate genotypes to the 95-32 cluster (MIRU profile 253335443432658253213423). This cluster is widely spread in Russia and is associated with MDR-TB, making it a significant risk factor for treatment failure or disease recurrence [15].

All isolates showed resistance to extended spectra of antituberculosis drugs. The first known isolate from Patient A, obtained in 2014, was already resistant to first-line drugs, fluoroquinolones, and kanamycin. The identified profile included the mutations *rpoB* Ser450Leu, *katG* Ser315Thr, *embB* Met306Val, *gyrA* Asp94Ala, and *eis* g(-37)t, according to the biochip test [16]. In downstream isolates analyzed via whole-genome sequencing, a continuous deletion including *pncA* 2278162-2289779 that ranged from *rv2030c* to *rv2045c* was found in 2015, resulting in pyrazinamide resistance.

### 2.2. Transmission Possibly Occurred in a Public Place

The contact tracing study of these patients revealed a long-lasting history of tuberculosis in the index case. Patient A was first diagnosed with tuberculosis in 1992. The patient had poor adherence to treatment, including self-discontinued therapy and refusal of surgical intervention, leading to the formation of a cavitary form of tuberculosis. After prolonged treatment with first-line drugs, he achieved clinical cure by 2002. However, in 2008, he experienced a disease relapse with newly acquired multi-drug resistance. Subsequent treatment failures, mainly caused by consistently poor compliance, resulted in the progression to extensively drug-resistant tuberculosis (fluoroquinolones plus second-line injectable drugs) with extensive fibrotic lung destruction and recurrent episodes of hemoptysis. During this course, culture conversion was not achieved, while the patient was engaged in high-risk transmission behaviors, including unrestricted use of public transportation and international air travel. Notably, HIV serology remained negative.

There was continuous amplification of drug resistance to first-line drugs and then sequentially to kanamycin, ethionamide, fluoroquinolones, PAS, and D-cycloserine. Finaly, resistance to the novel drugs bedaquiline and linezolid was developed. At the next exacerbation of the tuberculosis process with hemoptysis after leaving the hospital without permission, the patient died at home of pulmonary and heart failure against the backdrop of progressing tuberculosis in October 2019.

Patient B was a highly skilled intellectual worker suffering from an autoimmune disease of the hematopoietic system. He moved to Moscow from one of the regions of central Russia and lived in a rented apartment with his roommate in the same district as patient A. Patient B was diagnosed with tuberculosis in October 2019. After examination and treatment in general practice medical institutions, in January 2020, he was admitted to the Moscow Research and Clinical Center for Tuberculosis Control, where he was diagnosed with tuberculous meningoencephalitis. *M. tuberculosis* isolates were obtained from cerebrospinal fluid during lumbar puncture. Due to a wide spectrum of drug resistance, including resistance to linezolid, it was not possible to generate a complete treatment scheme. The tuberculosis process had a wave-like progressive course and, in July 2020, the patient died in the intensive care unit of the Moscow TB Center from brain edema caused by a tubercular process.

An analysis of the residence places of patient A and patient B, located 1.5 km from each other and equidistant from the nearest subway station, shows that they could have come into contact during transport or in one of the social infrastructure facilities located in the immediate vicinity of the subway station.

### 2.3. Genomic Variants Emerged During the Treatment and Transmission Event

During the treatment of the first patient, a large spectrum of different mutations in *rv0678* emerged; these are associated with an increase in the bedaquiline MIC from 0.06 to 0.5 mg/L on agar media. In isolate #1, analyzed via whole-genome sequencing (2015 year of isolation), the *rv0678* Glu113Lys substitution was present in 26% of sequencing reads, while the remaining 74% showed a frameshift mutation (Tyr145fs) in the same gene. More recent isolates had only *rv0678* Glu113Lys substitution at 100% allelic frequency with a simultaneous increase in the bedaquiline MIC to 0.5 mg/L (Figure 1), which is above the borderline value of 0.25 mg/L [17]. The *rplC* Cys154Arg, leading to linezolid resistance, emerged in 2015 and was stable in sequential isolates from Patient A, and was also found in isolates from Patient B.

In addition to the mutations in the *rv0678* gene associated with resistance to bedaquiline and *rplC,* associated with resistance to linezolid, other mutations emerged during the treatment of Patient A and the transmission of the strain. In total, 17 variants emerged or were transient during treatment and the transmission event, of which 11 resulted from the microevolution of the index case, 4 emerged during transmission, and 2 emerged during the short period of Patient B’s treatment.

For Patient A, the whole-genome analysis was conducted for isolates obtained during the four years between 2015 and 2019. Between the first and second isolate, separated by three years, four variants emerged and were fixed at 100% allelic frequency in *moeY*, PE_PGRS29, and *echA12* genes and the *hemN*-*rpfD* intergenic region. Another gene, *rhlE*, acquired transient Ser174Pro and Thr453fs mutations, where the frequency of the latter did not exceed 10% (Table 1).

During transmission, four other mutations emerged. One of them, synonymous amino acid substitution in the *rv1063c* gene encoding hydrolase, was probably neutral. The three others could have an impact on physiology and occurred in the PE/PGRS21 gene involved in autophagy inhibition during intracellular growth [18], RNA-degradosome component Rv2752c, and small RNA B11. In addition to these variants, the PpsA substitution Ala171Val was fixed at an allelic frequency of 100%, being in a mixed state in the index case, and the *rhlE* variants did not pass to the contact.

Two mutations emerged in the Patient B isolates after three months. While frameshift in *lldD2* at 100% frequency was certain, the second variant that occurred in the PE8-Rv1041c intergenic region was in an unannotated fragment of the *M. tuberculosis* genome of about 1000 bp in length; thus, no prediction of its impact could be made.

Of the 17 variants, 7 were loss-of-function variants caused by frameshifts or substitutions with the stop codons, and 2 were synonymous. Four genes—*rv2752c*, *ppsA*, *rhlE*, and *rv0678*—had two variants each, which could possibly indicate their importance for adaptation of the pathogen (Table 1).

## 3. Discussion

Our study confirmed the possibility of public transmission of the bedaquiline- and linezolid-resistant strain in the population, in line with previous observations of transmission of MDR strains [14,19]. It also provided evidence of the primary resistance of the pathogen to a wide spectrum of drugs, including bedaquiline [20,21]. An increasing rate of bedaquiline resistance in primary cases has been reported globally; the same is true for delamanid, which was introduced into clinical practice later [13,22]. Furthermore, bedaquiline resistance caused by loss-of-function mutations in *rv0678* (*mmpR5*) is particularly epidemiologically dangerous, as several new drugs share the same efflux mechanism of resistance. In this regard, the two candidate compounds BRD-8000 and BRD-9327, targeting two different sites in the essential EfpA pump, are both exported by MmpR5/S5 [23]. This is also true for various drugs targeting the arabinogalactans synthesis enzyme DprE1, which all are in phase 2 of clinical trials, including benzothiazinones (Macozinone) [24], Quabodepistat [25], and TBA-7371 [26].

Both social and molecular genetic properties play a role in the transmission of tuberculosis. We suspect that the success of the strain under study could be attributed mostly to the poor treatment adherence of Patient A. Termination of the treatment course occurred several times during about 20 years of history, accompanied with a stepwise increase in the pathogen resistance profile from susceptible to XDR, defined at present as resistance to fluoroquinolones and either bedaquiline or linezolid. Such cases call into question the idea of adaptability as an exclusive inherited property of specific *M. tuberculosis* lineages, omitting social aspects of infection.

The transmitted strain belongs to the Beijing Central Asian/Russian sublineage, which is known for its association with drug resistance. It is presumed to be more virulent, and rapid adaptation to treatment and host pressures is one of its possible properties [27]. It is worth noting that most of the bedaquiline- and linezolid-resistant isolates in Russia were from either Beijing B0/W148 or the Beijing Central Asian/Russian sublineages [28,29]. Moreover, most bedaquiline-resistant strains with AtpE substitutions also belong to these two sublineages [29].

We observed that 17 variants emerged or were lost during the microevolution and transmission event from 2015 and 2019, resulting in a mutation rate of 3.5 events per genome per year. To better fit the mutation rates estimated in previous reports, we omitted events in PE/PPE genes and transient events, which result in eight fixed variants emerging in 4.8 years. Thus, the resulting rate was 1.7 variants/genome/year, which is still noticeably higher than in previous reports [30]. Taking into account only microevolution events in isolates from the index case, where three variants have been fixed across 3.7 years, the mutation rate is estimated to be 0.8 variants/genome/year, which is closer to the average rate for *M. tuberculosis* (0.63) estimated in a systematic review [30] and equal to the reported 0.8 per genome per year calculated in the context of prolonged treatment in a single patient [31]. Mutation rates have been reported to differ for different lineages, and Lineage 2, which includes Beijing, is considered to mutate faster [32]. Thus, for the observed successful sublineage, it is not unexpected for this rate to be even higher.

Noticeably, bedaquiline resistance mutations in *rv0678* appeared early, following which the diversity decreased to the single variant, which was further transmitted. Amino acid substitution Glu113Ser led to MIC increases up to 1–2 mg/L (Bactec MGIT) and 0.5 mg/L (agar media), which are significantly higher than the wild-type corresponding modes of 0.125 mg/L for Bactec MGIT and 0.03 mg/L for agar media [9].

No other mutations in the genes previously associated with resistance to bedaquiline were found, while slight variability in the MIC values was observed. The direct target of the bedaquiline AtpE subunit was left unadjusted [33], as were AtpB [34] and PepQ [35]. Similarly, no mutations have been found in the more recently described *mmpL5* [29,36] and *mmpS4/L4* operon [29]. Poor adherence to treatment could be the main cause, as all additional variants emerged during prolonged treatment or serial passages used for the selection of mutants [36].

In the series of isolates from the first patient, the substitution C154R in RplC was the most frequent determinant of linezolid resistance found in clinical *M. tuberculosis* isolates [37]. *rv0678* and *rplC* are directly involved in resistance to bedaquiline and linezolid, respectively, and the transmitted strain was resistant and bore mutations in both genes.

Most of the other genome changes cannot be interpreted unambiguously, due to our imperfect knowledge of pathogen physiology. Aside from their possible neutral nature, the emergence of mutations could be driven by the selective pressure of the host, or could potentially compensate for the drug resistance fitness cost. Confirming the latter possibility, mutations in three genes—*rv2752c*, *ppsA,* and B11—were previously associated with resistance to various drugs.

Rv2752c encodes a bifunctional protein with RNase J activity and Zn^2+^-dependent metallo-β-lactamase activity. Homoplastic mutations in this gene were previously associated with resistance to rifampicin or isoniazid [38,39], ethionamide [40], and bedaquiline [41,42]. The frameshifting mutation in *ppsA* involved in PDIM biosynthesis was observed in both index and transmission cases at low allelic frequency; although this could also be a signature of resistance to pyrazinamide [43] or bedaquiline/linezolid [44], it is not clear how the pathogen copes with the loss of PDIM biosynthesis [43]. The small non-coding RNA gene B11 (ncRv13660c, MTS2822, C6) regulates *M. tuberculosis* transcripts associated with DNA replication (*dnaB*) and protein secretion (*eccA1*, *espE*, *espF*) [45], as does *panD*, the expression of which alters the susceptibility to pyrazinamide [46]. B11 sRNA affects colony morphology [47] and alters SDS sensitivity [48].

Mutations potentially associated with virulence traits include those in the *rhlE*, *echA12*, PE/PGRS21, and PE/PGRS29 genes. The component of RNA degradosome RhlE (Rv3211), which is an ATP-dependent RNA helicase, could have a general effect on various aspects of cell survival [49]. The deletion of 15 amino acids in PE_PGRS21 emerged during transmission. This gene was associated with the inhibition of autophagy during macrophage infection [18]. Interestingly, the in-frame insertion in this gene was found in a study of specific determinants of isolates obtained from tuberculosis meningitis patients with exactly the same diagnosis as Patient B [50].

The emergence of loss-of-function mutations in the *echA12* [51] and *lldD2* genes [52] shows that central metabolic pathways in the pathogen are also under selective pressure during infection.

Other variants, such as synonymous mutations in *rv1063c* and *moeY*, as well as substitutions in the intergenic regions PE8-*rv1041c* and *hemN*-*rpfD*, are probably neutral fixed variants that do not provide any inherent benefit or detriment to the survival of the mycobacterial cell.

In vitro selection and genome-wide association studies including identification of transmission clades provide the most important information on the drug resistance mechanisms and adaptation strategies of pathogens. In this context, studies of microevolution and transmission events are less informative, but they nevertheless show the exact evolutionary trajectories under the pressure of the treatment and the immune system. The development of resistance is not necessarily associated with fitness cost, or could be rapidly compensated for by secondary mutations [53,54]. This study demonstrates the high risk of transmission of XDR-TB strains in urban settings and highlights the need to carefully monitor not only known resistance-associated mutations but also genotypes, in order to track the spread of TB.

## 4. Materials and Methods

### 4.1. Clinical Isolates

Clinical isolates were obtained from Patients A and B at the Moscow Research and Clinical Center for Tuberculosis Control. For Patient A, we analyzed five isolates collected between 2015 and 2019 after unsuccessful treatment with a bedaquiline–linezolid regimen. For patient B, we analyzed three isolates with resistance to linezolid and bedaquiline, collected between 2019 and 2020.

Drug susceptibility testing for rifampicin, isoniazid, streptomycin, ethambutol, pyrazinamide, ofloxacin, moxifloxacin, kanamycin, capreomycin, amikacin, PAS (para-aminosalicylic acid), and ethionamide was performed using the Bactec MGIT 960 system (Becton Dickinson, Franklin Lakes, NJ, USA), as described previously [17]. The critical concentrations for kanamycin, amikacin, and capreomycin were 2.5 mg/L, 1.0 mg/L, and 2.5 mg/L, respectively.

We performed bedaquiline and linezolid susceptibility testing using a modified proportional method on the Bactec MGIT 960 system. Russian national guidelines for tuberculosis treatment align with WHO recommendations, and we used the currently approved critical concentrations for bedaquiline and linezolid (1 mg/L for both drugs). For bedaquiline, we prepared the drug by dissolving and diluting it in DMSO and adding 100 μL per MGIT tube. Linezolid (Glenmark Pharmaceuticals, Mumbai, Maharashtra, India) was dissolved in sterile water, as recommended in the guidelines.

Genomic DNA isolation, amplification, and Sanger sequencing of the *rplC* and *rv0678* fragments were performed as previously described [3].

### 4.2. MIRU-VNTR Typing

Genotyping at 24 MIRU-VNTR loci was performed as described previously using agarose gels [55]. Profiles in the article are given in the following order: MIRU04 (ETRD-1), MIRU26, MIRU40, MIRU10, MIRU16, MIRU31 (ETRE), Mtub04, ETRC, ETR-A, Mtub30, Mtub39, Qub4156, Qub11b, Mtub21, Qub26, MIRU02, MIRU23, MIRU39, MIRU20, MIRU24, MIRU27 (Qub5), Mtub29, ETRB, Mtub34. The MIRU profiles were compared online to MIRU-VNTR plus (http://www.miru-vntrplus.org/; accessed on 31 July 2025).

### 4.3. Whole-Genome Sequencing and Bioinformatic Analysis

Strains for whole-genome sequencing were recultured on Lowenstein–Jensen media for 3 to 4 weeks at 37 °C and then underwent heat inactivation. Genomic DNA was isolated using the Qiagen Gentra Puregene Yeast/Bact. Kit (cat no. 158567, QIAGEN, Venlo, The Netherlands) with minor modifications: prolonged incubation with lysozyme for 4 h, and optional chloroform–isoamyl alcohol purification after the protein precipitation step.

DNA libraries were prepared using an Illumina DNA Prep kit and sequencing was performed using the MiniSeq High Output Kit (300 cycles) on the MiniSeq platform (Illumina, San Diego, CA, USA).

The sequencing data in FastQ format were analyzed using the Galaxy web platform (https://usegalaxy.org, last accessed on 1 February 2025). The reads were trimmed using the Trimmomatic tool (ver. 0.39), mapped to the *M. tuberculosis* H37Rv reference genome (GenBank accession NC_000962.3) with BWA-MEM2 (ver. 2.3), and were refined using BamLeftAlign (ver. 0.0.2). Variant calling was performed using FreeBayes (ver. 1.3.10) with default parameters and filtered with the VCFlib toolkit (ver. 1.0.0_rc3) with the (QUAL > 20) option. Variant annotation was performed using SnpEff (ver. 5.4). Further bioinformatic analysis was performed by joining all variants in an Excel table and visual inspection of BAM files using the UGENE software (ver. 53.1).

Since the alignment of short reads to the reference resulted in low-confidence data on repetitive sequences, we reanalyzed the raw sequence data using the genomic sequence of the *M. tuberculosis* isolate 2-0034P6C4 (RefSeq assembly GCF_014900175.1), which is genetically closer to the studied isolates [56]. The coverage of the alignment was more uniform, and the list of variants found in all studied isolates was shortened to 280 compared to 2478 found when strain H37Rv genome was used as reference (Figure S1). Two genomes were aligned using Mauve (ver. 2.4.0), and minor rearrangement near the 330,500 position was identified (Figure S1). The custom Python script ‘vcfvisual’ was used for visualization of variants along the reference genomes.

Highly repetitive genomic loci were dropped from the variant analysis based on visual inspection of BAM files and comparison of variant qualities between isolates (Tables S1 and S2). In total, 188 genes and 71 intergenic fragments comprising about 8% of the genome were excluded for *M. tuberculosis* H37Rv. For the second reference genome, 33 genes and 31 intergenic regions (about 2% of the genome) were proposed to contain improperly aligned reads and false positive variants. Thus, 156 high-quality variants were identified when the *M. tuberculosis* str. 2-0034P6C4 sequence was used as a reference.

Long deletion of 11,617 base pairs between positions 2278162 and 2289779 (H37Rv numbering), which included the *pncA* gene associated with pyrazinamide resistance, was identified via visual inspection of the BAM files using the UGENE software (Figure S1).

## Figures and Tables

**Table 1:** All microevolution events identified via whole-genome sequencing in sequential isolates from patients A and B. Allelic frequencies of variants are shown as a percentage of reads with mutation to a total number of reads at this position.

		Patient A					Patient B		
		#1 Bc.104	#2 Bc.110	#3 Bc.111	#4 Bc.112	#5 Bc.113	#1 Js.001	#2 Js.002	#3 Js.003
Rv0678	Glu113Lys	26%	100%	100%	100%	100%	100%	100%	100%
Rv0678	Tyr145fs	74%							
PE_PGRS29	Gly124Arg		100%	100%	100%	100%	100%	100%	100%
HemN	C-96T		100%	100%	100%	100%	100%	100%	100%
MoeY	Leu536Leu		100%	100%	100%	100%	100%	100%	100%
EchA12	Trp237stop		100%	100%	100%	100%	100%	100%	100%
RhlE	Ser174Pro		20%	80%	90%	90%			
RhlE	Thr453fs			10%					
Rv1063c	Arg27Arg						100%	100%	100%
PE_PGRS21	Val259_Gly273del						100%	100%	100%
Rv2752c	Pro390fs						100%	100%	100%
Rv2752c	Leu355fs			10%					
B11	cag86cagg						100%	100%	100%
PpsA	Ala171Val						100%	100%	100%
PpsA	Glu958fs	10%				10%	10%	10%	90%
PE8-Rv1041c	-661 A>G								100%
IldD2	Ile279fs								100%

## Data Availability

All raw sequence reads were submitted to the National Center for Biotechnology Sequence Read Archive database and are available under accession no. PRJNA768108. Strain IDs in the database are the same as those used in Table 1. The vcf file visualization script is available in the GitHub repository, https://github.com/DanZimenkov/ngs_tools (accessed on 11 May 2026).

## Supplementary Materials

The following supporting information can be downloaded at: https://www.mdpi.com/article/10.3390/ijms27114912/s1.
