# Supplementary material for: A Case of Transmission of Bedaquiline- and Linezolid-Resistant Tuberculosis
Source: Int J Mol Sci. 2026 May 29;27(11):4912. doi: 10.3390/ijms27114912 (PMC13256819; doi:10.3390/ijms27114912)
Supplement: Supplementary file 1 [file ijms-27-04912-s001.zip › ijms-4241405-supplementary.pdf]

Supplementary data

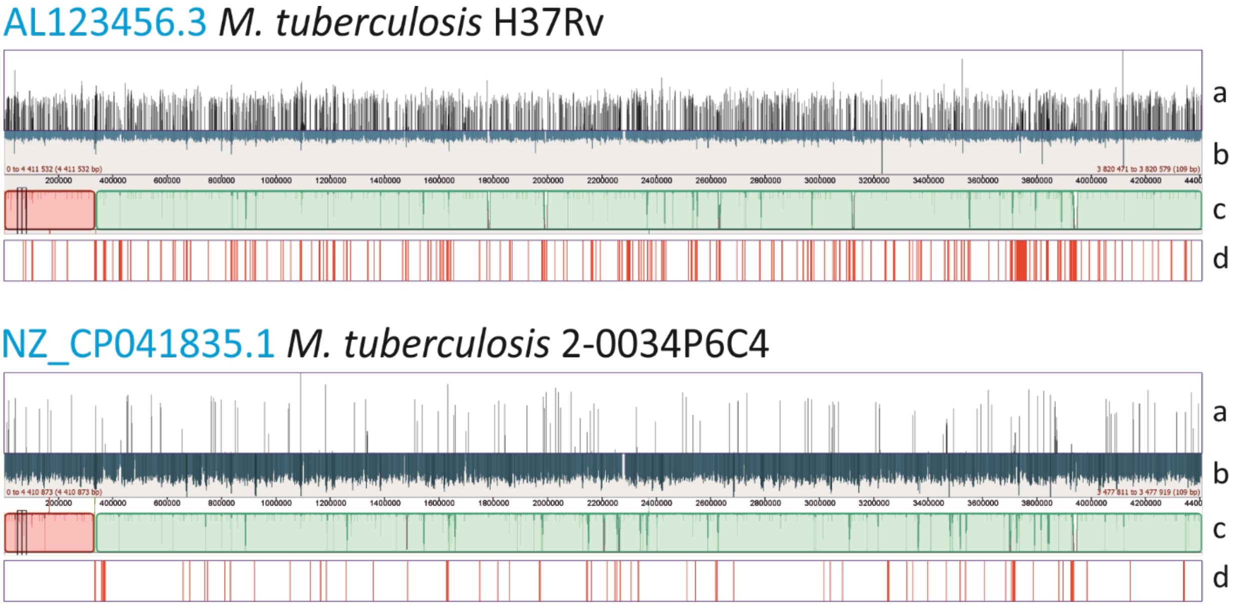

Figure S1. Alignment and variant calling using two reference genomes.

The conventional *M. tuberculosis* H37Rv (lineage 4) and *M. tuberculosis* str. 2-0034P6C4 (lineage 2, Central Asia/Russia genotype) genomes were used;

(a) – plotted variants along the genome. Line heights are proportional to QUAL parameter of vcf file;

(b) – bam file coverage screenshot from UGENE software;

(c) – Mauve alignment of two genomes;

(d) – location of genes with repetitive sequences dropped from the variant analysis.

Table S1. List of genes and intergenic regions in *M. tuberculosis* H37Rv genome containing highly repetitive sequences.

Locus	Start	End
Rv0064-Rv0064A	71559	71589
Rv0071	79486	80193
Rv0094c	103710	104663
Rv0094c-Rv0095c	104663	104805
Rv0095c	104805	105215
Rv0096	105324	106715
Rv0151c	177543	179309
Rv0159c	187433	188839
Rv0197	232231	234519
Rv0278c	333437	336310
Rv0278c-Rv0279c	336310	336560
Rv0279c	336560	339073
Rv0280	339364	340974
Rv0304c	366150	372764
Rv0305c	372820	375711
Rv0336	400192	401703
Rv0354c	424269	424694
Rv0355c	424777	434679
Rv0379-Rv0380c	456192	456268
Rv0387c	466672	468001
Rv0442c	530751	532214
Rv0487	576787	577338
Rv0490	579349	580581
Rv0532	622793	624577
Rv0538	630040	631686
Rv0570-Rv0571c	663373	663487
Rv0578c	671996	675916
Rv0589	686821	688035
Rv0655-Rv0656c	752596	752984
Rv0724	815663	817534
Rv0746	835701	838052
Rv0747	838451	840856
Rv0754-Rv0755c	847913	848103
Rv0759c-Rv0760c	854157	854267
Rv0766c	858864	860072
Rv0794c-Rv0795	888636	889072
Rv0797	890388	891482
Rv0814c	908181	908483
Rv0823c	916477	917646
Rv0829	921575	921865
Rv0833	925361	927610

Rv0872c	968424	970244
Rv0915c	1020058	1021329
Rv0922	1027685	1029337
Rv0946c-Rv0947c	1056685	1057300
Rv0976c-Rv0977	1090175	1090373
Rv0978c	1093361	1094356
Rv0978c-Rv0979c	1094356	1094670
Rv0980c	1095078	1096451
Rv0980c-Rv0981	1096451	1096822
Rv1013-Rv1014c	1133259	1133333
Rv1037c	1160544	1160828
Rv1040c	1162549	1163376
Rv1040c-Rv1041c	1163376	1164572
Rv1041c	1164572	1165435
Rv1054	1176928	1177242
Rv1067c	1188421	1190424
Rv1068c	1190757	1192148
Rv1087	1211560	1213863
Rv1089	1214769	1215131
Rv1091	1216469	1219030
Rv1128c	1251617	1252972
Rv1135c	1262272	1264128
Rv1148c	1276300	1277748
Rv1149	1277893	1278300
Rv1196	1339349	1340524
MTB000075	1340578	1340625
Rv1198	1341006	1341290
Rv1199c	1341358	1342605
Rv1219c-Rv1220c	1363361	1363503
Rv1243c	1384989	1386677
Rv1313c	1468171	1469505
Rv1318c	1479199	1480824
Rv1319c	1480894	1482501
Rv1324-Rv1325c	1488075	1488154
Rv1325c	1488154	1489965
Rv1361c	1532443	1533633
Rv1387	1561769	1563388
Rv1396c	1572127	1573857
Rv1418-Rv1419	1593325	1593505
Rv1430	1606386	1607972
Rv1435c	1612342	1612950
Rv1441c	1618209	1619684
Rv1450c	1630638	1634627
Rv1452c	1636004	1638229
Rv1459c	1644363	1646138

Rv1468c	1655609	1656721
Rv1548c	1751297	1753333
Rv1572c	1779194	1779298
Rv1588c	1789168	1789836
Rv1626-Rv1627c	1828797	1828865
Rv1651c	1862347	1865382
Rv1658-Rv1659	1872559	1872639
Rv1705c	1931497	1932654
Rv1753c	1981614	1984775
Rv1754c	1984979	1986670
Rv1759c	1989833	1992577
Rv1765A-Rv1766	1998515	1999737
Rv1793	2030694	2030978
Rv1818c	2061178	2062674
Rv1829-Rv1830	2074437	2074841
Rv1883c	2133231	2133692
Rv1917c	2162932	2167311
Rv1918c	2167649	2170612
Rv1929c	2181262	2181906
Rv1945	2195989	2197353
Rv2015c	2261816	2263072
Rv2020c	2266421	2266720
Rv2023A	2268268	2268726
Rv2030c	2276441	2278486
Rv2048c	2294531	2306986
Rv2061c-Rv2062c	2317085	2317169
Rv2081c	2338065	2338505
Rv2082	2338709	2340874
Rv2090	2347373	2348554
Rv2098c	2356729	2358033
Rv2112c	2370905	2372569
Rv2123	2381071	2382492
Rv2126c	2387202	2387972
Rv2141c-MTB000025	2401722	2401987
Rv2162c	2423240	2424838
Rv2166c-Rv2167c	2429858	2430159
Rv2168c-Rv2169c	2431420	2431565
Rv2177c	2439282	2439947
Rv2247-Rv2248	2522164	2522360
Rv2258c	2530836	2531897
Rv2261c	2534042	2535552
Rv2263	2535641	2536594
Rv2274c	2546488	2546805
Rv2276	2547749	2548939
Rv2280	2551560	2552939

Rv2281-Rv2282c	2554831	2554938
Rv2328	2600731	2601879
Rv2346c	2625888	2626172
Rv2347c	2626223	2626519
Rv2352c-Rv2353c	2634098	2634528
Rv2355-Rv2356c	2636889	2637688
Rv2356c	2637688	2639535
Rv2402-Rv2403c	2700457	2700535
Rv2424c	2720776	2721777
Rv2434c	2729115	2730560
Rv2478c-Rv2479c	2784608	2784657
Rv2480c-Rv2481c	2785918	2786575
Rv2512c	2828556	2829803
Rv2519	2835785	2837263
Rv2543	2866468	2867127
Rv2544	2867124	2867786
Rv2591	2921551	2923182
Rv2615c	2943600	2944985
Rv2615c-Rv2616	2944985	2945330
Rv2634c	2960105	2962441
Rv2647-Rv2648	2972027	2972160
Rv2649-Rv2650c	2973421	2973795
Rv2650c	2973795	2975234
Rv2652c-Rv2653c	2976554	2976586
Rv2666	2983071	2983874
Rv2710-Rv2711	3023432	3023565
Rv2741	3053914	3055491
Rv2747-Rv2748c	3059786	3059855
Rv2752c	3064515	3066191
Rv2769c	3078158	3078985
Rv2770c	3079309	3080457
Rv2795c-Rv2796c	3104911	3105056
Rv2804c	3112465	3113094
Rv2807	3113658	3114812
Rv2812	3116818	3118227
Rv2820c	3127364	3128272
Rv2823c	3129344	3131773
Rv2825c	3132892	3133539
Rv2828c	3135788	3136333
Rv2853	3162268	3164115
Rv2885c	3194166	3195548
Rv2931	3245445	3251075
Rv2940c	3276380	3282715
Rv2979c	3335164	3335748
Rv2980-Rv2981c	3336505	3336796

Rv2991-MTB000039	3348473	3348547
Rv3003c-Rv3004	3362986	3363348
Rv3010c-Rv3011c	3369854	3369950
Rv3018c	3376939	3378243
Rv3021c	3379376	3380452
Rv3022c	3380440	3380682
Rv3053c-Rv3054c	3414958	3415435
Rv3097c	3465778	3467091
Rv3109	3477649	3478728
Rv3115	3481451	3482698
Rv3136	3501794	3502936
Rv3144c	3510088	3511317
Rv3159c	3527391	3529163
Rv3169	3537238	3538362
Rv3183-Rv3184	3551044	3551281
Rv3187-Rv3188	3554025	3554298
Rv3191c-MTB000043	3558345	3559370
Rv3192-Rv3193c	3560024	3560194
Rv3247c-Rv3248c	3628063	3628160
Rv3281	3663689	3664222
Rv3303c-Rv3304	3690938	3691141
Rv3319-Rv3320c	3707563	3707642
Rv3324A-Rv3325	3710379	3710433
Rv3326-Rv3327	3711694	3711749
Rv3327	3711749	3713461
Rv3328c	3713394	3714332
Rv3329	3714392	3715708
Rv3338-Rv3339c	3724548	3724615
Rv3343c	3729364	3736935
Rv3344c	3736984	3738000
Rv3345c	3738158	3742774
Rv3347c	3743711	3753184
Rv3349c	3754293	3755237
Rv3350c	3755952	3767102
Rv3367	3778568	3780334
Rv3379c-Rv3380c	3794867	3795100
Rv3387	3800786	3801463
Rv3388	3801653	3803848
Rv3388-Rv3389c	3803848	3803919
Rv3401-Rv3402c	3820402	3820653
Rv3423c-Rv3424c	3841420	3841714
Rv3424c	3841714	3842076
Rv3424c-Rv3425	3842076	3842239
Rv3425	3842239	3842769
Rv3425-Rv3426	3842769	3843036

Rv3426	3843036	3843734
Rv3426-Rv3427c	3843734	3843885
Rv3427c-Rv3428c	3844640	3844738
Rv3428c	3844738	3845970
Rv3428c-Rv3429	3845970	3847165
Rv3429	3847165	3847701
Rv3466	3883525	3884193
Rv3467	3883964	3884917
Rv3473c-Rv3474	3890733	3890830
Rv3475-Rv3476c	3892091	3892371
Rv3478	3894426	3895607
Rv3490-Rv3491	3909738	3909890
Rv3507	3926569	3930714
Rv3508	3931005	3936710
Rv3511	3939617	3941761
Rv3511-Rv3512	3941761	3943812
Rv3512	3943812	3944963
Rv3514	3945794	3950263
Rv3515c	3950824	3952470
Rv3532-Rv3533c	3970563	3970705
Rv3558	3997980	3999638
Rv3590c	4031404	4033158
Rv3595c	4036731	4038050
Rv3611	4052950	4053603
Rv3619c	4059984	4060268
Rv3620c	4060295	4060591
Rv3653	4093940	4094527
Rv3680	4119795	4120955
Rv3711c-Rv3712	4156729	4156981
Rv3750c-Rv3751	4198597	4198874
Rv3785	4231320	4232393
Rv3798	4252993	4254327
Rv3826	4299812	4301566
Rv3827c	4301563	4302789
Rv3872	4350745	4351044
Rv3876	4353010	4355010
Rv3878	4356693	4357535
Rv3892c	4374484	4375683

Table S2. List of genes and intergenic regions in *M. tuberculosis* str. 2-0034P6C4 genome containing highly repetitive sequences.

Locus	Start	End
FPJ73_RS20555	334770	337511
FPJ73_RS01565	359772	361544
FPJ73_RS01600	364585	374130
FPJ73_RS02975-FPJ73_RS02980	661793	661907
FPJ73_RS03070	685235	686449
FPJ73_RS03395-FPJ73_RS03400	740912	741118
FPJ73_RS03435-FPJ73_RS03440	751544	751932
FPJ73_RS03785-FPJ73_RS03790	814632	814734
FPJ73_RS03920	834705	837059
FPJ73_RS21205-FPJ73_RS21210	924060	924961
FPJ73_RS04970-FPJ73_RS20630	1055025	1055640
FPJ73_RS05330-FPJ73_RS05335	1131612	1131686
FPJ73_RS05500	1167777	1169024
FPJ73_RS05615	1189129	1190667
FPJ73_RS05975	1262801	1264000
FPJ73_RS06390-FPJ73_RS06395	1363235	1363377
FPJ73_RS06955-FPJ73_RS06960	1489471	1489550
FPJ73_RS07620	1632002	1636198
FPJ73_RS07630	1637575	1639980
FPJ73_RS08140	1754458	1756494
FPJ73_RS08460-FPJ73_RS08465	1822711	1822779
FPJ73_RS08625-FPJ73_RS08630	1866494	1866574
FPJ73_RS09115	1975821	1979363
FPJ73_RS09905-FPJ73_RS09910	2150607	2150842
FPJ73_RS09915	2152152	2153624
FPJ73_RS09975	2167575	2168219
FPJ73_RS21255-FPJ73_RS10350	2225020	2225109
FPJ73_RS10495	2254473	2255734
FPJ73_RS10540	2260429	2261106
FPJ73_RS10585	2273601	2275646
FPJ73_RS10735-FPJ73_RS10740	2314245	2314329
FPJ73_RS10860-FPJ73_RS10865	2340116	2340517
FPJ73_RS10870	2341760	2342754
FPJ73_RS11715-FPJ73_RS11720	2520877	2521001
FPJ73_RS11875-FPJ73_RS11880	2551424	2551531
FPJ73_RS12270	2624718	2625377
FPJ73_RS12290	2629843	2631690
FPJ73_RS12540-FPJ73_RS12545	2692612	2692690
FPJ73_RS14225-FPJ73_RS14230	3026684	3026820
FPJ73_RS14355-FPJ73_RS14360	3049179	3049248
FPJ73_RS14595-FPJ73_RS14600	3094304	3094449

FPJ73_RS15330	3260123	3266458
FPJ73_RS15595-FPJ73_RS15600	3332337	3332411
FPJ73_RS15705-FPJ73_RS15710	3353718	3353814
FPJ73_RS15980-FPJ73_RS15985	3409780	3409897
FPJ73_RS16325	3477112	3478287
FPJ73_RS16565	3527768	3528892
FPJ73_RS16675	3547801	3548025
FPJ73_RS16685-FPJ73_RS16690	3549196	3549366
FPJ73_RS16990-FPJ73_RS16995	3617242	3617333
FPJ73_RS17360-FPJ73_RS17365	3696659	3696738
FPJ73_RS17465-FPJ73_RS17470	3717444	3717511
FPJ73_RS17490	3722260	3731601
FPJ73_RS17705-FPJ73_RS17710	3798661	3798732
FPJ73_RS18190	3892352	3893533
FPJ73_RS18250-FPJ73_RS18255	3907665	3907817
FPJ73_RS18355	3936707	3938859
FPJ73_RS21325	3939107	3939885
FPJ73_RS21330	3941222	3942454
FPJ73_RS18365	3944043	3948745
FPJ73_RS18590	3996461	3998119
FPJ73_RS19380-FPJ73_RS19385	4156071	4156323
FPJ73_RS20255	4352312	4354312
FPJ73_RS20265	4355995	4356837
